# Mapping of prostate cancer microvascular patterns using super-resolution ultrasound imaging

**DOI:** 10.1186/s41747-025-00561-6

**Published:** 2025-02-20

**Authors:** Mairead B. Butler, Georgios Papageorgiou, Evangelos D. Kanoulas, Vasiliki Voulgaridou, Hessel Wijkstra, Massimo Mischi, Christophe K. Mannaerts, Steven McDougall, William Colin Duncan, Weiping Lu, Vassilis Sboros

**Affiliations:** 1 Heriot-Watt University, Institute of Biological Chemistry, Biophysics and Bioengineering, Engineering and Physical Sciences, Edinburgh, EH14 4AS UK; 2 Eindhoven University of Technology, Electrical Engineering, Eindhoven, The Netherlands; 3 Department of Urology, Radboud University Medical Center, Nijmegen, The Netherlands; 4 The Centre for Reproductive Health, Institute for Regeneration and Repair, 4-5 Little France Drive, Edinburgh BioQuarter, Edinburgh, EH16 4UU UK

**Keywords:** Algorithms, Animals, Microbubbles, Prostatic neoplasms, Ultrasonography

## Abstract

**Background:**

Super-resolution ultrasound imaging (SRUI) is a rapidly expanding field with the potential to impact cancer management. Image processing algorithms applied to contrast-enhanced ultrasound (CEUS) video data can track the path of the contrast agent and produce high-resolution maps of vascular networks. Our aim was to develop SRUI for mapping prostate vascular dynamics and to assess the feasibility of identifying vascular patterns associated with prostate cancer.

**Methods:**

Tracking algorithms for SRUI were developed using *in silico* data and validated in pre-clinical CEUS video collected from the sheep ovary. Algorithm performance was then assessed in a retrospective study of 54 image planes within 14 human prostates. CEUS data was collected for each plane, and regions of suspected cancer in each were identified from biopsy data.

**Results:**

Of three algorithms assessed, utilising vascular knowledge was found to be the most robust method. Regions of suspected cancer were associated with increased blood flow volume and speed while avascular regions were also identified. Ten scan planes had confirmed Gleason 7 cancer; of these 10 planes, 7 had distinct regions of fast and high-volume flow, while 6 had both avascular and high flow regions. The cancer-free planes had more consistent, low blood flow values across the plane.

**Conclusion:**

SRUI can be used to identify imaging biomarkers associated with vascular architecture and dynamics. These multiparameter biomarkers may be useful in pinpointing regions of significant prostate cancer.

**Relevance statement:**

Super-resolution ultrasound imaging can generate microvascular maps of the prostate, revealing tissue patterns and presenting significant potential for the identification of multiple biomarkers associated with the localisation of prostate cancer.

**Trial registration:**

Retrospectively registered NCT02831920, date 5/7/2016 https://www.clinicaltrials.gov/study/NCT02831920.

**Key Points:**

- An algorithm was developed and tested in synthetic pre-clinical and clinical data.
- Maps of blood vessels were created using contrast-enhanced ultrasound imaging.
- Specific presentations of vasculature at regions of prostate cancer have been identified.

**Graphical Abstract:**

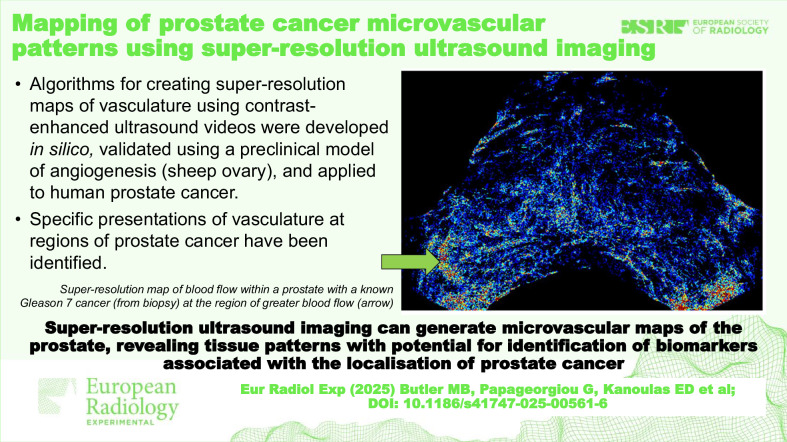

## Background

Prostate cancer is one of the highest-incidence cancers worldwide [1] and the second most common cause of cancer death in men. Histopathological evaluation of prostate cancer (PCa) shows variation in the presentation of the cancer within the gland [2, 3**]**. Therefore, the ease of detection and diagnosis of PCa can depend on the type of cancer cells as well as the location of the lesion within the prostate, in a gland that changes with age [4, 5**]**.

Multiparametric magnetic resonance imaging (mpMRI) includes T2-weighted, diffusion-weighted and dynamic contrast-enhanced sequences, providing a sensitivity of over 90% in PCa detection [6]. This sensitivity is dependent on the experience of the reporting clinician. The sensitivity for clinically significant PCa has been reported to be 85% for uroradiologists compared to 70% for abdominal radiologists, with an overall accuracy of 80% and 54%, respectively [7]. In addition, while technology is improving [8], the specificity of mpMRI in PCa diagnosis remains low, indicating a fundamental limitation in the detection of the disease [9]. As a result, mpMRI leads to a large number of unnecessary biopsies, leading to high costs for healthcare providers and unnecessary invasive procedures for patients [10]. Further, as PCa is a multifocal disease, systematic biopsy is opted for alongside targeted biopsy as lesions outside the targeted space are common [11]. It is recognised that mpMRI does not identify all prostate lesions, as some are invisible to MRI [12]. In addition, mpMRI can be time-consuming, costly and is not readily available to all patients.

Multiparametric ultrasound imaging has been proposed as an alternative option to mpMRI for prostate imaging [13]. The combination of B-mode, Doppler and contrast-enhanced ultrasound (CEUS) imaging along with elastography has been shown to be effective in PCa diagnosis, slightly inferior in sensitivity to mpMRI, and was proposed by Grey et al as a reasonable alternative to mpMRI [13]. The authors concluded that there is potential for ultrasound imaging to be placed in the prostate imaging pathway, particularly for patients who cannot tolerate MRI or where MRI is not readily available. It is recognised that ultrasound technologies are more accessible, cost-effective and portable, and generally represent an effective healthcare choice. In addition, high-frequency and micro-ultrasound have also been proposed as options for prostate imaging [14]. However, this approach is limited in imaging the prostate transition zone.

State-of-the-art clinical imaging used in the detection and staging of cancer, including MRI and ultrasound imaging, provides a macroscopic and mostly semiquantitative assessment of vascular perfusion [6]. A key limitation lies in the achievable resolution. A number of different vessel sizes are likely to be present within a ‘resolution cell’ (*i.e*., the voxel or pixel) and as a result the image captures a cumulative intensity effect, which, for CEUS, is not linear [15] and is dominated by larger vessels, making the assessment of microvascular activity highly challenging. No single vascular-derived biomarkers have shown strong sensitivity or specificity in the diagnosis of PCa [16–18]. For example, the association of microvascular density with the tumour stage remains inconclusive: a number of studies showed an increased microvascular density in malignant tissue compared to benign, while others showed no link [16]. While angiogenesis is essential for the growth of PCa, similar to many tumours, imaging of angiogenesis is limited by the resolution of current macroscopic techniques [16, 18, 19]. Contrast-enhanced imaging within the mpMRI protocol is not primarily used in assigning PI-RADS scores [20], suggesting that a detectable increase in blood flow is not a leading biomarker in PCa. In addition, increased blood flow has been recorded in PCa compared to prostates with no cancer [21], suggesting a potential limitation with current imaging methods. It is likely that improved resolution and recording of blood flow may help develop biomarkers for PCa.

Super-resolution ultrasound imaging (SRUI) is different from high-frequency and micro-ultrasound imaging. It is achieved by processing CEUS mode video data offline to localise and track the injected microbubbles tracer, in a way that ‘paints’ their paths within the lumen of vessels within the imaging plane [22]. These maps have a higher image resolution compared to traditional ultrasound imaging. SRUI shows microscale detail, providing a ‘direct’ and near-histological view of the prostate structure in terms of functional vessels and their dynamics. However, while several SRUI methodologies demonstrate application *in silico* [23] or *in vitro* [24], very few perform well *in vivo* [25], particularly using state-of-the-art imaging equipment on human subjects [26], which have lower frame rates and frequencies. A key challenge is the robustness of the microbubbles tracking, which can miss existing vessels or produce erroneous ones.

This work aims to provide a new approach to microbubble tracking. The developed models will be applied to *in silico* computational data before applying to pre-clinical data to undertake essential validation. This will be followed by applying the models to unblinded clinical data to assess the feasibility of SRUI for the detection of PCa and distinguishing flow patterns and features in diseased prostates.

## Methods

**Fig. 1:**
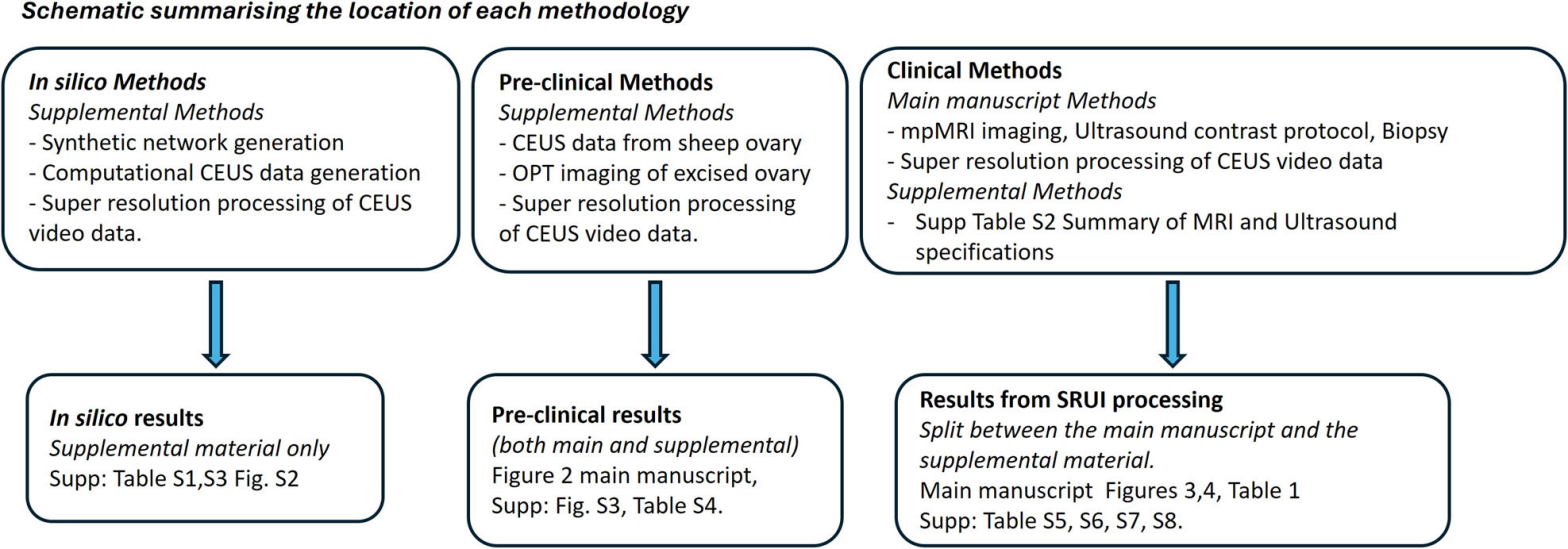
Schematic diagrams describing the *in silico*, pre-clinical and clinical data pathways and their location within the associated documentation. CEUS, Contrast-enhanced ultrasound; MRI, Magnetic resonance imaging; OPT, Optical projection tomography; SRUI, Super-resolution ultrasound imaging

Pre-clinical work was conducted under the United Kingdom home office licence Duncan PPL 60/4401 and clinical data under IRB approval METC AMC 2015_263, Clinicaltrials.gov (NCT02831920). The methodology followed essential incremental steps to ensure validation of the algorithm: *in silico*, *in vivo* pre-clinical, and clinical experiments. Figure 1 presents a schematic describing the three method pathways and details the location of the methods and results for each. The computational *in silico* work enabled the validation of microbubbles localisation and tracking. The pre-clinical work involved CEUS imaging of the ovine corpus luteum (CL), which provided tissue morphology and vessel architecture validation as well as being an initial feasibility study to assess performance in real life data. The CL is a structure, which is known to comprise dense microvessels representing well the tumour microenvironment [27]. Further, the structure of the CL presents microvessels surrounded by larger vessels, with large feeder vessels located outside, thus allowing the full range of vessel sizes to be distinctly captured within one imaging location. Thus, the entire vascular bed is ideally situated for mapping a single SRUI plane. This validation, enhanced by comparison to optical projection tomography (OPT), gave confidence that SRUI produces outputs showing real vascular and microvascular structures. Finally, the clinical data assessed viability in a clinical setting and enabled the assessment of tumour location with biopsy as confirmation of diagnosis. The *in silico*, and pre-clinical methods are fully described in the Supplemental Material, while the development of the SRUI tracking methodology and application to clinical data is described here.

### SRUI method

The SRUI processing of CEUS data comprised two main parts: (1) detection and localisation of microbubble echoes within the CEUS images; and (2) association and linking of the detected microbubbles in consecutive frames. For the detection of microbubbles, all methods presented in this work follow the procedure described by Kanoulas et al [22]: the microbubbles were detected, segmented, and localised according to the intensity-weighted centre of mass. Tracking microbubbles is generally efficient in very high frame rate modes (0.1–100 kHz), due to microbubbles image coherence in consecutive frames. While research ultrasound scanners offer high frame rate capability [28, 29], state-of-the-art clinical equipment utilise lower frame rates (5–30 Hz) in CEUS mode, which makes the tracking particularly challenging.

The primary goal of the tracking was to avoid the generation of false tracks that create erroneous vessels in the final SRUI output. Three tracking approaches were assessed: (a) the nearest neighbour (NN) method [22] uses the nearest echo in the subsequent frame to establish the link between detections; (b) forward model uses a forward motion predictive filter to reject arbitrary microbubbles jumps and unnatural directions of travel such as backward motion; and (c) a vascular model (VM) which in addition to forward model includes criteria based on vascular characteristics. In particular, links with average microbubble path densities that are significantly different at the start and end of the track or links whose microbubble path density has a large deviation from its average value are rejected. These additional criteria in the VM ensure that tracked microbubbles are within the vascular space and not creating false links across different vessels, an example of true and false tracks is given in the Supplemental Material (Supplementary Fig. S1).

### Clinical study overview

A retrospective study was undertaken with CEUS data collected from 14 men with a suspicion of PCa. The participants had been referred for prostate imaging and biopsy as part of a prospective study at the Amsterdam UMC, Amsterdam, the Netherlands (METC AMC 2015_263-Clinicaltrials.gov NCT02831920). The additional data collection for the study here did not require a significant change to the original protocol. The selection criteria were dictated by the primary clinical trial, no adjustments in selection were made for the purpose of this manuscript. The primary clinical trial included biopsy-naïve men with an elevated serum prostate-specific antigen level (≥ 3.0 ng/mL) and/or suspicious digital rectal examination in the Amsterdam UMC, Amsterdam, the Netherlands, between December 2015 and November 2018. Men were excluded if they had a prostate-specific antigen level > 20 ng/mL, a digital rectal examination suggestive of extracapsular disease or contra-indications for mpMRI and/or CEUS. The trial design was consistent with the Standards of Reporting for MRI-Targeted Biopsy Studies (START) recommendations [30]. The primary trial comprised 150 participants. A subset of 14 participants, for the work presented here, underwent additional acquisition of CEUS video data, which involved an additional period of 3 min acquisition per image plane per injection. These 14 participants, numbered from P122 to P135, were those scheduled for the study at the time of the additional data collection. The inclusion criteria of the primary clinical trial were the only selection criteria applied. This feasibility study was designed for a small subgroup as it was deemed appropriate in order to initially assess the algorithms in clinical data with a view to designing a future study.

### Ultrasound imaging

For ultrasound imaging, scanning was performed by two urologists. Both of whom had two years’ experience of transrectal ultrasound imaging of the prostate and had performed over 100 prostate ultrasound procedures per year as well as at least 50 prostate biopsy procedures per year. They also received two months of hands-on training in CEUS of the prostate beforehand with an additional three months of supervised CEUS scans.

The participants were in lateral decubitus position, and their prostate was scanned in up to four planes: base, mid-base, mid-apex and apex, using an iU22 ultrasound scanner with C10-3V transrectal transducer (Philips Medical Systems, Bothell, WA, USA). The scanner was run in contrast imaging mode using a power modulation sequence at 3.5 MHz, with a low mechanical index (0.06) to prevent the destruction of the microbubbles. Imaging frame rates used ranged from 9 to 11 Hz. In total, from all 14 cases, 54 scan planes were collected, and each one was assessed independently. For each scan plane a bolus injection of 2.4 mL SonoVue (Bracco, Geneva, Switzerland), was administered, the total dose per participant was 9.6 mL SonoVue (the recommended clinical dose per bolus is 2 mL for cardiac imaging and 2.4 mL for vascular Doppler imaging [31]). The first two min of the bolus injection (the wash-in to peak intensity frames) were used in the primary clinical trial. For the study here, the following three min of video data, where the contrast had passed its peak intensity, was saved on the ultrasound scanner, transferred as a DICOM file, and later processed offline.

### mpMRI

Prior to biopsy, the participants underwent mpMRI on either a 1.5-T INGENIA® MR System (Philips Medical Systems, Best, The Netherlands) or a 3-T MAGNETOM® Avanto (Siemens Healthcare, Erlangen, Germany) MRI scanner, using a pelvic phased array coil. Full MRI specifications are provided in the supplemental material (Supplemental Table S2) [32]. The MRI scanner used in each case is described in Supplemental Table S8.

### Systematic biopsy

After ultrasound scanning and mpMRI, a 12-core systematic transrectal biopsy was undertaken along with a targeted biopsy (using MRI fusion or cognitive targeting to the output from the primary clinical trial) where deemed necessary. All biopsy cores were examined by one dedicated uropathologist with ≥ 12 years’ of experience. The systematic biopsy (two cores collected from the apex and base and four cores each from the mid-apex and mid-base) [32] provided information on the location of the positive biopsy cores within the prostate, however for most cases, the actual size of any lesion was not known.

As a result of the clinical diagnoses received post-biopsy most of the participants included in this study did not follow the path of radical prostatectomy therefore, as also seen in the literature, the reference standard associated with our comparisons is the systematic biopsy [13]. The clinical reporting from mpMRI and biopsy was available to the authors and used to validate the SRUI result in each image slice.

### Ultrasound video data processing

The SRUI image analysis algorithms were used to process the *in silico*, pre-clinical, and clinical data. After running all three algorithm versions, *i.e*., NN, forward model, VM, their performance was evaluated in terms of tracks, microbubble detections, track length (links per track), and microbubble speed. The outputs led to super-resolved maps with the number of formed tracks, microbubble velocity, and blood flow (the product of track number and velocity) of the scanned organ. In the clinical data, features within the super-resolution maps at regions of suspected cancer (from biopsy diagnosis) were identified and compared with regions of assumed normal tissue. Full numerical assessments were undertaken in two cases where small regions of interest (ROI) in the region of diagnosed cancer were compared to an ROI of the same size, assumed to contain no cancer cells (from biopsy) within the same prostate imaging slice. Statistical significance between populations was assessed by *t*-test. For small data sets, normality was assessed by the Shapiro-Wilks test, and for larger data sets (*n* > 5,000) normality was assumed due to the central limit theorem.

## Results

The SRUI algorithms were developed and assessed *in silico* prior to pre-clinical evaluation. This staged technological development was important to provide confidence in the final clinical results, where the histological validation only refers to PCa grade. The following section details the main findings from the pre-clinical and clinical data. Results from *in silico* work, comparing the performance of the three different algorithms are presented in the Supplemental material (Supplemental Fig. S2 and Table S3).

### Pre-clinical data

**Fig. 2:**
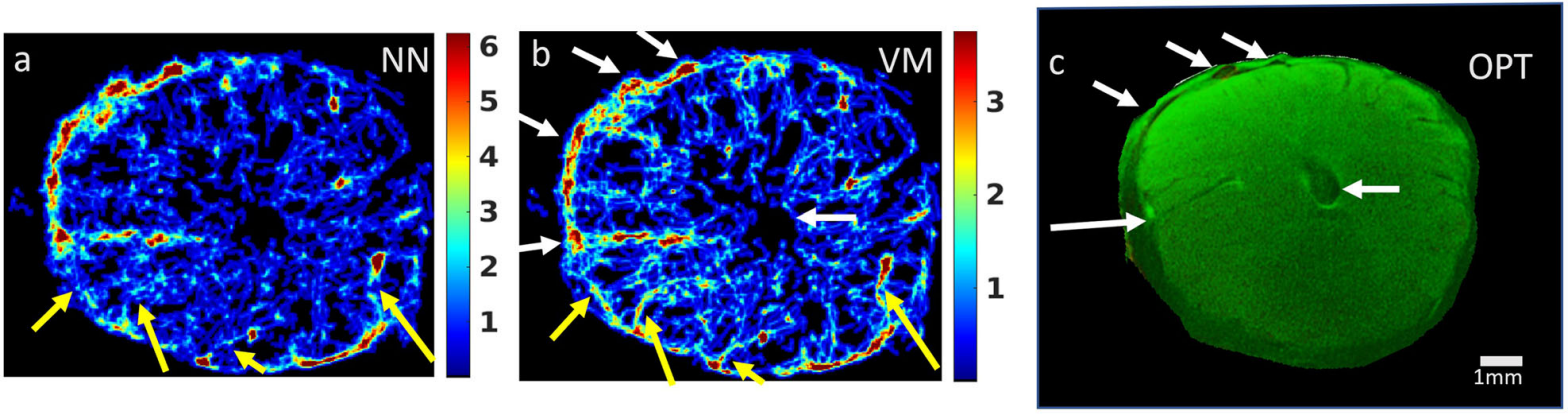
Track number maps (smoothed number of tracks per pixel) from contrast-enhanced ultrasound video data from a sheep corpus luteum. Video data was processed using both nearest neighbour (NN) (**a**) and vascular model (VM) (**b**) tracking algorithms. **c** shows the corresponding corpus luteum cross-section from optical projection tomography (OPT) of the whole sheep ovary with white arrows identifying similar regions in each. Yellow arrows indicate regions where disconnected links within the NN processing are seen as linked regions (complete vessels) with the VM processing

Figure 2 shows the SRUI track number map of the CL from a sheep ovary using NN (panel a) and VM (panel b) models. The well-known structure of the CL comprises larger vessels on the outer edge, with narrower vessels, less than 200 µm diameter, in the central region. This structure is seen in Fig. 2a, b with higher track density (red), indicating larger vessels (white arrows) on the outer edge and vessels containing fewer tracks in the central region. When comparing the SRUI maps to the OPT of the same cross-section, the large vessels surrounding the CL and the central cyst region comprising no blood vessels (Fig. 2, white arrows) were seen in both the ultrasound maps (panels a and b) and OPT image (panel c). The qualitative comparison shows defined vessels reconstructed by the VM model that are missing or interrupted in the NN model (yellow arrows). Quantitative outputs and further comparison between the models are given in the supplemental material (Supplemental Fig. S3 and Table S4).

### Prostate cancer imaging

Of the 14 men, 5 were diagnosed with PCa by biopsy and 9 had benign tissue. In total, 54 imaging planes were independently assessed and biopsy information for each plane was used to determine whether clinically significant cancer (Gleason 3 + 4 = 7 and above) was present. Clinical diagnoses spanning no PCa, T1c, T2c and T3a were found. Ten of the 54 imaging planes had confirmed Gleason 3 + 4 = 7 or above. One case (4 image slices) had no known diagnosis due to the absence of biopsy (P126). None of the cases included in the study had positive lymph nodes or metastases. SRUI outputs from the imaging planes with confirmed cancer were assessed to identify any vascular characteristics that could potentially be associated with cancer, some specific case examples which highlight different SRUI features seen are given below.

#### Example of highly angiogenic cancer (P127)

**Fig. 3:**
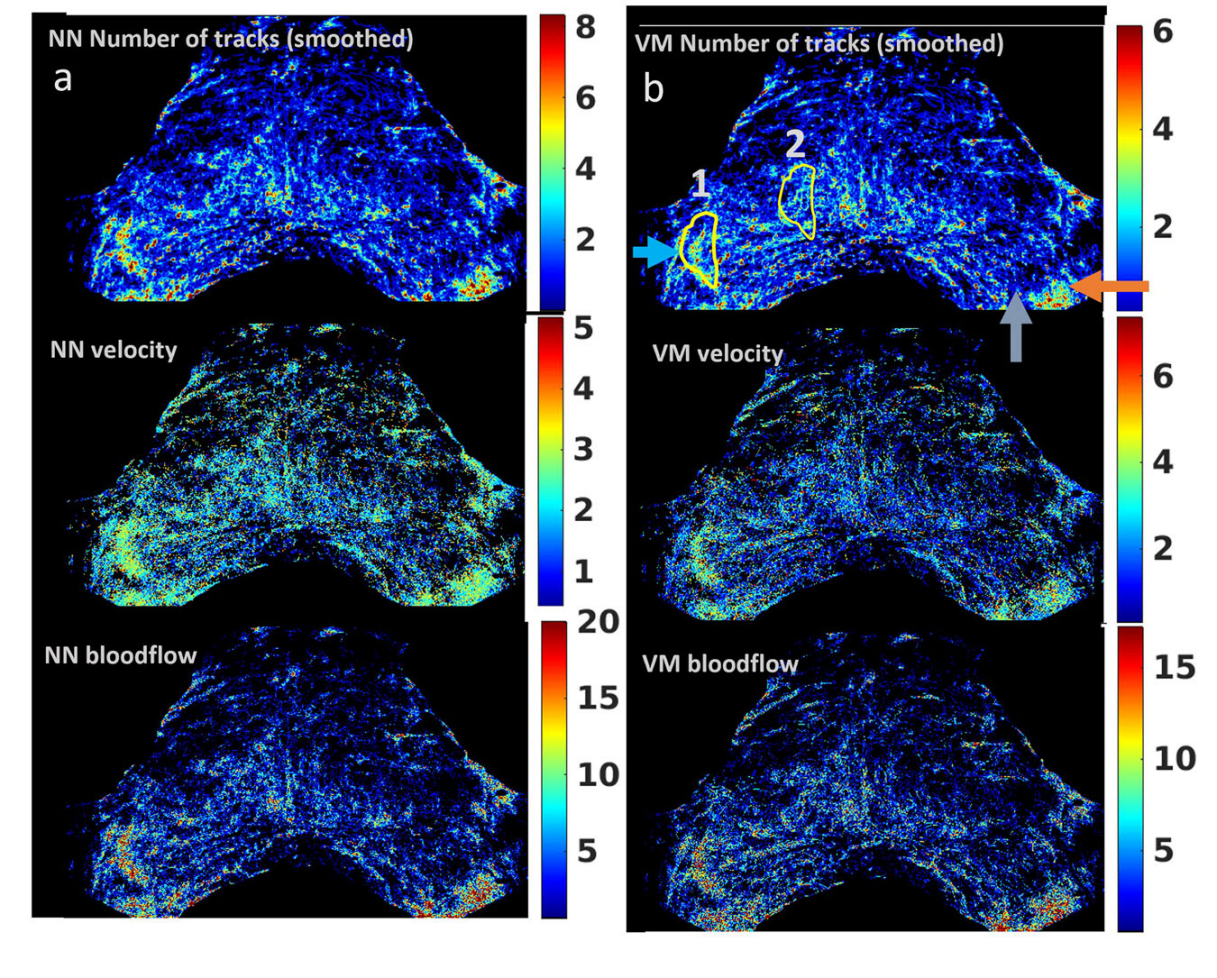
Data from contrast-enhanced ultrasound of the prostate (participant 127, mid-apex plane). Nearest neighbour (NN) (**a**) and vascular model (VM) (**b**) processing was applied to the video data, and super-resolution ultrasound imaging maps showing a number of tracks per pixel, velocity and blood flow were created. Arrowed regions are those where there is known cancer (blue) and a region of cancer, but there is uncertainty in the boundary of the prostate (grey and orange), the region associated with higher blood flow on this side may be linked to a cancer region or maybe the neurovascular bundle outside of the prostate. The VM model maps were formed from more tracks than the NN model, but those tracks were created from fewer links. A table of outputs is provided in the Supplemental material (Table S5). The two drawn regions (1 and 2) were processed independently, and the outputs for healthy and diseased tissue were compared. Region 1 (the cancer region) was found to have a higher mean velocity compared to region 2 (*p* < 0.001), as well as more detections and shorter tracks (Table S6)

Initial output from SRUI processing for P127 in the mid-apex image plane is given in Fig. 3. This case was classified as PI-RADS 5, T2c. At this imaging location, the systematic biopsy identified PCa (Gleason 7) at left lateral and right lateral locations (unidentified on MRI). SRUI maps (number of tracks, velocity and blood flow) were created by NN (Fig. 3a) and VM (Fig. 3b) tracking algorithms. Higher velocities were measured, and more tracks were created comprising fewer links using the VM compared to the NN. While similar main features (high flow and low flow regions) were seen in both NN and VM processing, NN is highly likely to provide false tracks, and therefore, the VM model is best suited for assessing clinical data. A highly vascularised region (Fig. 3b, blue arrow) is deemed to correspond with the cancer region identified by targeted biopsy. Although it is known that systematic biopsy found a Gleason 7 tumour tissue on the left, a similar region of well-defined increased blood flow within the prostate was not clearly seen. However, in this case, the left boundary of the prostate is harder to define. A highly vascularised region (orange arrow) is seen at the extreme left, but it is not clear if any part of this vascular region is within the prostate border. Further, there is a region of low flow at the lateral left prostate (grey arrow) which may also be associated with the tumour. Outputs assessing different smaller ROIs within this prostate are given in Table 1 where the cancer region (Fig. 3b, ROI 1) had faster mean velocity (4.1 ± 2.0 mm/s) compared to the cancer-free region (Fig. 3b, ROI 2) (3.1 ± 1.9 mm/s) (*p* < 0.001). Differences were also seen in the number of tracks and blood flow of the cancer and cancer-free regions (*p* < 0.001). Numerical outputs from both algorithm versions are available in the supplemental material (Tables S5 and S6).

**Table 1:** Velocities calculated by super-resolution ultrasound imaging for known cancer and assumed non-cancer regions within P127

	Nearest neighbour, ROI cancer 1	Vascular model, ROI cancer 1	Nearest neighbour, ROI no cancer 2	Vascular model, ROI no cancer 2 (*p* < 0.001 comparing to velocity model ROI 1)
Number of frames processed	1,008	1,008	1,008	1,008
Detected microbubbles	11,672	11,672	6,916	6,916
Mean velocities (standard deviation) (mm/s)	3.6 (1.1)	4.1 (2.0)	2.8 (1.3)	3.1 (1.9)
Median velocities (quartiles 25% and 75%) (mm/s)	3.7 (2.9, 4.3)	3.8 (2.6, 5.3)	2.7 (1.8, 3.6)	2.6 (1.6, 4.0)

The cancer and non-cancer regions are presented in Fig. 2

#### Example of nonangiogenic cancer (P134)

**Fig. 4:**
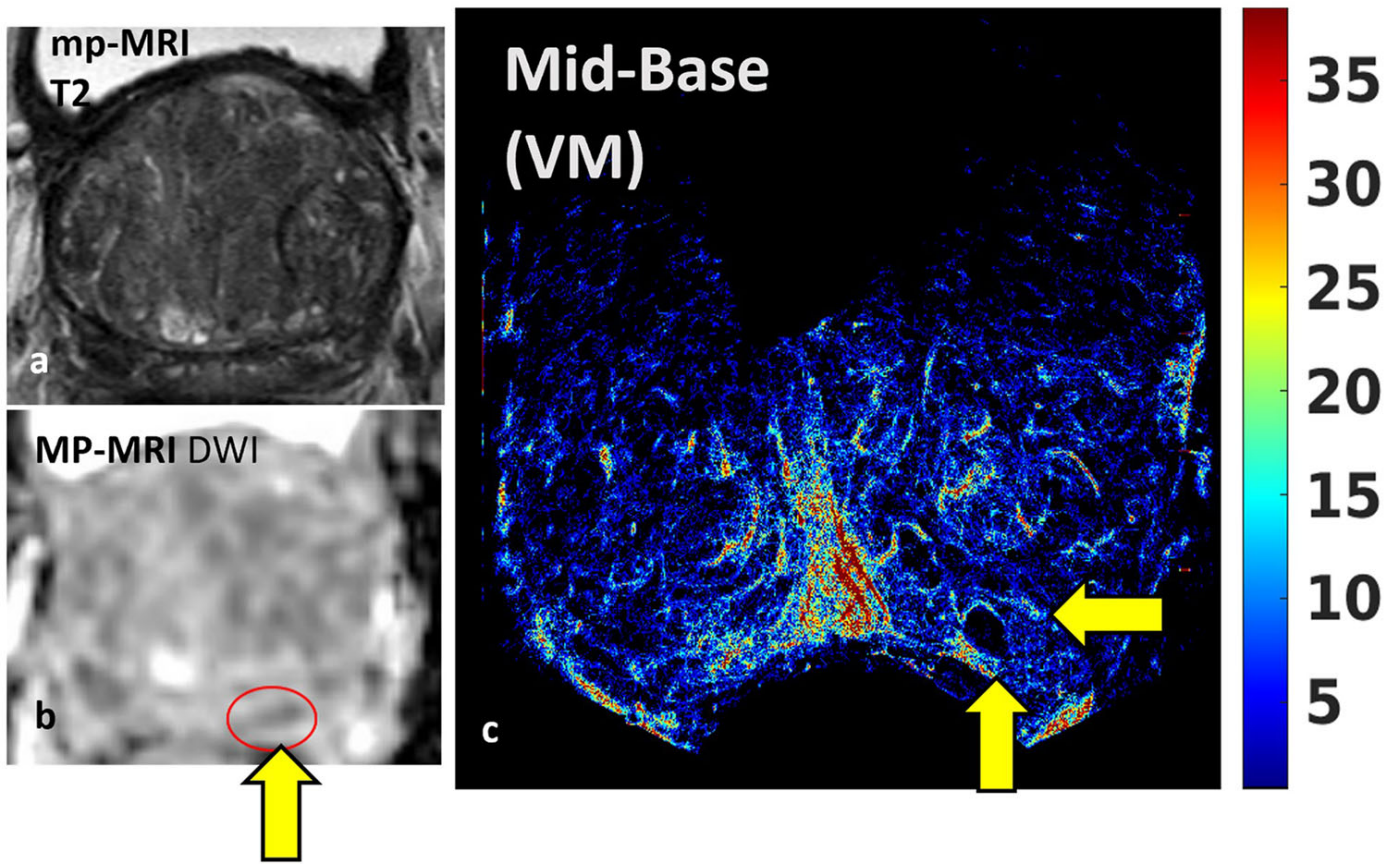
Multiparametric magnetic resonance imaging (mpMRI) compared with blood flow super-resolution ultrasound map for a prostate where the cancer was found by biopsy in the left posterior area. **a** T2-weighted image for the mid-base slice in Participant 134 (T2cNxMx PI-RADS 4); **b** diffusion-weighted image where a yellow arrow shows the region of suspicion; **c** blood flow super-resolution ultrasound mid-base map by vascular model (VM). While mpMRI (and biopsy) found a suspicious lesion in the mid-prostate in the left peripheral zone (**b**), in the super-resolution blood flow map, we see an avascular region alongside a smaller region of increased flow on either side (yellow arrows). A table containing the number of tracks and links is provided in the supplemental material (Table S7). As with previous data sets, the VM model provides a map containing fewer false tracks, and more correct tracks, created from fewer and shorter links

Figure 4 shows MRI outputs (panels a and b) and the blood flow map (panel c) from the mid-base area of participant 134, where systematic biopsy produced a negative result, however, mpMRI diagnosis was T2c, PI-RADS 4 [33] (1 lesion noted by diffusion restriction on MRI, peripheral zone, maximum 8 mm in length at left mid posterior medial region (arrowed)). Further targeted biopsy (based on output from the primary CEUS study) found Gleason 3 + 4 = 7 at the left lateral and medial posterior mid-base slice. The presentation on the SRUI map (Fig. 4c) shows an avascular region separated by areas of higher blood flow compared to the surrounding tissue (yellow arrow). The avascular region may potentially be ischaemic, hypoxic, cystic or may be attributed to glandular prostate tissue comprising no functioning vasculature. P134 (Fig. 4) provided a different numerical result compared to P127 as the cancer region had similar velocity to an equivalent cancer-free region (2.2 ± 1.4 and 2.4 ± 1.5 mm/s, respectively) as well as similar blood flow (6.4 ± 6.7 and 6.6 ± 6.6 tracks.mm/s, respectively) (Supplemental Table S7).

#### Clinical data summary

A full summary of all 54 planes of patient data is given in Supplemental Table S8. Of the 10 imaging planes with confirmed Gleason 7 cancer, 9 contained avascular regions, 7 had regions of fast and dense flow, while 6 had both avascular and high flow regions. Participants with no known cancer had predominantly low flow velocities in sparse distributions of vessels which appears to differentiate them from participants with Gleason 7 cancer.

## Discussion

A novel SRUI tracking algorithm that uses CEUS video data acquired using state-of-the-art clinical equipment is presented here. The output was near-microscopic detailed maps of prostate vascular architecture and dynamics, including avascular regions that have low or no flow, thus presenting a functioning/dynamic histology-like display of the prostate. Vascular patterns across the whole prostate cross-section (not limited to the peripheral zone) could be visualised. In a total of 54 independent image planes (from 14 patients), the specific features associated with Gleason score 7 cancer included high vessel density and velocity as well as avascular domains. These results strongly suggest that SRUI has potential for imaging PCa and the hypotheses of new SRUI biomarkers will be tested in a future prospective clinical study, which will aim to formulate a SRUI-driven multiparametric ultrasound scoring system along with an AI-assisted decision-making tool.

Multiparametric ultrasound has been shown to have potential in PCa diagnostics only being slightly inferior to mpMRI [13]. As a tool, CEUS imaging can already provide information on perfusion and flow parameters [34–36]. However, due to the resolution and the non-linearity of the contrast signal, the larger vessels within the imaging region dominate the measurements [15, 35, 37, 38]. SRUI is a departure from macroscopic visualisation and provides a mapping of the vascular and microvascular architecture and flow dynamics within the imaged region and could potentially provide additional sensitivity and specificity in multiparametric ultrasound, making it competitive to mpMRI.

It is important to note that some normal or benign highly vascularised regions also exist within the prostate. For example, the normal increased flow of main arteries around the urethra and the transition zone is typically more vascular than the peripheral. Further, conditions such as benign prostatic hyperplasia or prostatitis are recognised as being associated with variations in blood flow. Therefore the limited sensitivity of CEUS or dynamic contrast-enhanced imaging is also attributed as a limitation in differentiating these other conditions from tumour-driven angiogenesis [34, 36]. The SRUI maps here show potential for the depiction of complex vascular patterns with variable degrees of vascular and avascular activity as a result of a lesion within the gland, thus enabling differentiation between normal and abnormal tissue with the potential to offer increased diagnostic specificity. The example cases (P127 and P134) highlight the potential for variability in the presentation of cancer in SRUI outputs while also indicating that there may be quantifiable differences in the outputs from regions of cancer compared to those with no cancer. Other than vessel density and the direct measurement of velocity, biomarkers could represent size, shape and other vessel structural and dynamic patterns or arrangements. In comparison, conventional MRI and ultrasound imaging, depict a strong signal from a single tissue property (such as blood flow, tissue stiffness, acoustic impedance and water density) over a reasonably large volume of several cubic millimetres. We, thus, hypothesise that the significant number of patients with PCa who would not be identified using conventional imaging may present a more subtle and complex tissue property pattern, not identifiable at the mm scale in a single tissue property measurement. Indeed, the variability observed in the vascular patterns of the tumours here may explain the mpMRI invisibility of some PCas [39], as several physical properties may combine at the near-microscale and thus be averaged out at the mm resolution of MRI.

A larger patient study may address this hypothesis and enable the identification of tissue patterns, mapped by the vascular architecture and dynamics associated with different prostate conditions. In fact, the current study is not an assessment of the diagnostic accuracy of SRUI but an investigation of its potential for detecting PCa. Since the SRUI maps depict previously unseen pathophysiological details, following the successful identification of tissue patterns associated with cancer in the future, we would then seek to clarify the clinical position in the prostate patient management pathway, and whether it could potentially replace or operate alongside MRI, guide targeted biopsy, be used in patient surveillance or plan and monitor therapy.

The specificity and positive predictive values in detecting significant cancer would require a different study design, more clinical data, particularly given the vascular appearance of a number of prostate conditions. The limited number of cases with PCa in this study meant it was not possible to fully assess the appearance of cancers in different zones of the prostate. Cancers in the transition zone may have different vascular characteristics to those in the peripheral zone. However, the SRUI processing can be undertaken in all prostate zones. Therefore, features specific to different cancer types in different prostate locations may emerge as further clinical data is acquired and imaging protocols optimised. Given the emerging complexity that can be captured in the SRUI maps, multiparametric biomarkers or complex patterns are likely to be candidate biomarkers for PCa detection. This may require a larger number of case studies compared to what would be the case for conventional imaging.

The technical advance here offered increased confidence in the maps provided by the specific VM tracking model and is backed by reliability in recovering true links and the avoidance of generating erroneous links leading to false vessel reconstruction. This robustness is built from *in silico* development and was validated in the pre-clinical CL data where microvessels (*i.e*., arterioles) can be identified reliably and in accordance with histological evaluation. It is recognised that the current version of the VM model may require further improvement in order to utilise as many of the detected microbubbles as possible.

In conclusion, SRUI has been developed and assessed *in silico*, tested in pre-clinical data and applied to clinical data sets to provide maps of prostate vascularisation in near-microscopic detail. We have shown that prostate SRUI is feasible. It was found that an SRUI tracking method using vascular knowledge can show tumorous domains with variable vascular architecture and dynamics patterns, the next step being to address hypotheses on the clinical utility of this technology.

## Supplementary information

**Additional file 1: Fig. S1.** Zoomed in density map of the clinical data set. The black star is the initial microbubble (MB) location in frame t and white-centre star is the MB location at frame t+1. The line between them corresponds to paired MBs when no rejection criteria are applied. The black circle corresponds to a correct link within a high MB density region (indicates a vessel), whereas the links circled in white correspond to incorrect pairing with MBs “jumping” from one vessel to the next passing through a low MB density region, *i.e.*, inconsistent density within the formed “track”. **Fig. S2** (**a**) The simulated network structure, (**b**) an example individual frame of the synthetic contrast-enhanced ultrasound video data (**c**) The microbubble speed maps calculated from the synthetic data set using nearest neighbour (NN), forward model (FM) and vascular model (VM) versions of the tracking algorithm. (**d**) Average intensity profile through the selected region in part (**c**) for each processing method. Each vessel is better resolved in the VM version compared to the NN and FM versions. (**e**) scatter plot comparing average full-width half maximum (FWHM) for each peak in part (**d**). FM (circles) and VM (x) methods are compared to NN (x-axis). The FWHM calculated by NN and FM models are very similar (mean NN 179.6 ± 25 μm mean FM 179.0 ± 26 μm) (*p* = 0.5), following y = x, while the VM consistently model produces narrower vessels (mean 140.7 ± 16 μm) (*p* = 0.001). **Fig. S3.** Displaying 25–90% of the peak intensity value from the track number maps of the central Corpus Luteum (CL) region (see Figure 2) for Nearest Neighbour (NN) and Vascular model (VM) methods. The central CL is known to comprise very small vessels. The VM version of the code creates more links within this region and shows vessels that are likely to be between 200 and 50 μm in diameter. The NN version produces a few tracks and covers an area of 34 mm_2_. The area covered inside the CL with VM is 82.2 mm_2_, showing an increase of 137%. **Table S1** The specifications of the computational synthetic Vascular Network (detailed Figure S2 panel a). A tube-diameter range wider than 1.5 mm is provided, which corresponds to tubes with realistic blood speeds ranging between 3.8 and 23.87 mm/sec. **Table S2** MRI and Ultrasound specifications[12] Abbreviations: AT = acquisition time, mpMRI = multiparametric magnetic resonance imaging, TR = Repetition Time, TE = Echo Time, FOV = Field of View. *(Invivo, Gainesville, FL, USA). **Table S3** Synthetic data. Outputs comparing the detections and links made by 3 different versions of the tracking algorithm: Nearest Neighbour (NN), Forward Model (FM), Vascular Model (VM). **Table S4** Comparison of detection and linking outputs from contrast-enhanced ultrasound video data from the sheep corpus luteum (CL) for nearest neighbour (NN) version and the Vascular model (VM) version. **Table S5** Quantitative output from Nearest Neighbour (NN) and Vascular Model (VM) tracking models from participant 127 Mid-Apex (Fig.3 main document). **Table S6** Processing outputs for P127 vascular model (VM) and nearest neighbour (NN) processing for cancer and non-cancer regions (ROI 1 and 2 in Fig 3). **Table S7** Output for both nearest neighbour (NN) and vascular model (VM) tracking models for P134 (Fig 4 main document). Also included are the numerical outputs (VM) from smaller regions of interest for cancer and non-cancer regions of the mid-base plane. **Table S8** Full summary of all clinical prostate data processing using super-resolution ultrasound imaging. Each data set that has been processed is represented, and those where unusual regions of low, high or fast flow were noted (x). Entries in **bold** are associated with a clinical diagnosis of Gleason 7 cancer by biopsy. The super-resolution method appears to have good sensitivity in that areas of faster flow can be identified and these are consistently associated with regions of cancer, further work is needed to fully assess the specificity and identify regions of the prostate where faster flow is normal.

## Data Availability

The data sets generated and/or analysed during the current study are not publicly available but may be available from the corresponding author upon reasonable request. Algorithms used for the processing of the data will not be available due to protected intellectual property.

## Abbreviations

CEUS: Contrast-enhanced ultrasound
CL: Corpus luteum
mpMRI: Multiparametric magnetic resonance imaging
MRI: Magnetic resonance imaging
NN: Nearest neighbour
OPT: Optical projection tomography
PCa: Prostate cancer
ROI: Region of interest
SRUI: Super-resolution ultrasound imaging
VM: Vascular model
