# Supplementary material for: Mapping of prostate cancer microvascular patterns using super-resolution ultrasound imaging
Source: Eur Radiol Exp. 2025 Feb 20;9:25. doi: 10.1186/s41747-025-00561-6 (PMC11842657; doi:10.1186/s41747-025-00561-6)
Supplement: Supplementary file 1 — Additional file 1: Fig. S1. Zoomed in density map of the clinical data set. The black star is the initial microbubble (MB) location in frame t and white-centre star is the MB location at frame t+1. The line between them corresponds to paired MBs when no rejection criteria are applied. The black circle corresponds to a correct link within a high MB density region (indicates a vessel), whereas the links circled in white correspond to incorrect pairing with MBs “jumping” from one vessel to the next passing through a low MB density region, i.e., inconsistent density within the formed “track”. Fig. S2 (a) The simulated network structure, (b) an example individual frame of the synthetic contrast-enhanced ultrasound video data (c) The microbubble speed maps calculated from the synthetic data set using nearest neighbour (NN), forward model (FM) and vascular model (VM) versions of the tracking algorithm. (d) Average intensity profile through the selected region in part (c) for each processing method. Each vessel is better resolved in the VM version compared to the NN and FM versions. (e) scatter plot comparing average full-width half maximum (FWHM) for each peak in part (d). FM (circles) and VM (x) methods are compared to NN (x-axis). The FWHM calculated by NN and FM models are very similar (mean NN 179.6 ± 25 μm mean FM 179.0 ± 26 μm) (p = 0.5), following y = x, while the VM consistently model produces narrower vessels (mean 140.7 ± 16 μm) (p = 0.001). Fig. S3. Displaying 25–90% of the peak intensity value from the track number maps of the central Corpus Luteum (CL) region (see Figure 2) for Nearest Neighbour (NN) and Vascular model (VM) methods. The central CL is known to comprise very small vessels. The VM version of the code creates more links within this region and shows vessels that are likely to be between 200 and 50 μm in diameter. The NN version produces a few tracks and covers an area of 34 mm2. The area covered inside the CL with VM is 82.2 mm2, showing an increas [file 41747_2025_561_MOESM1_ESM.pdf]

Mapping of prostate cancer microvascular patterns using super-resolution ultrasound imaging

ELECTRONIC SUPPLEMENTARY MATERIAL

Fig. S1. Zoomed in density map of clinical dataset. The black star is the initial microbubble (MB) location in frame t and white-centre star is the MB location at frame t+1. The line between them corresponds to paired MBs when no rejection criteria are applied. The black circle corresponds to a correct link within a high MB density region (indicates a vessel), whereas the links circled in white correspond to incorrect pairing with MBs “jumping” from one vessel to the next passing through a low MB density region, i.e., inconsistent density within the formed “track”.

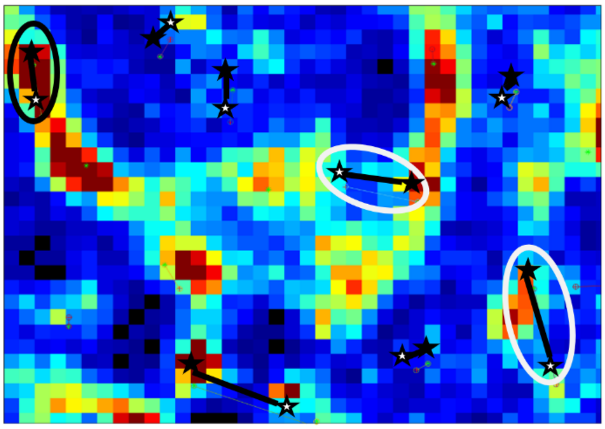

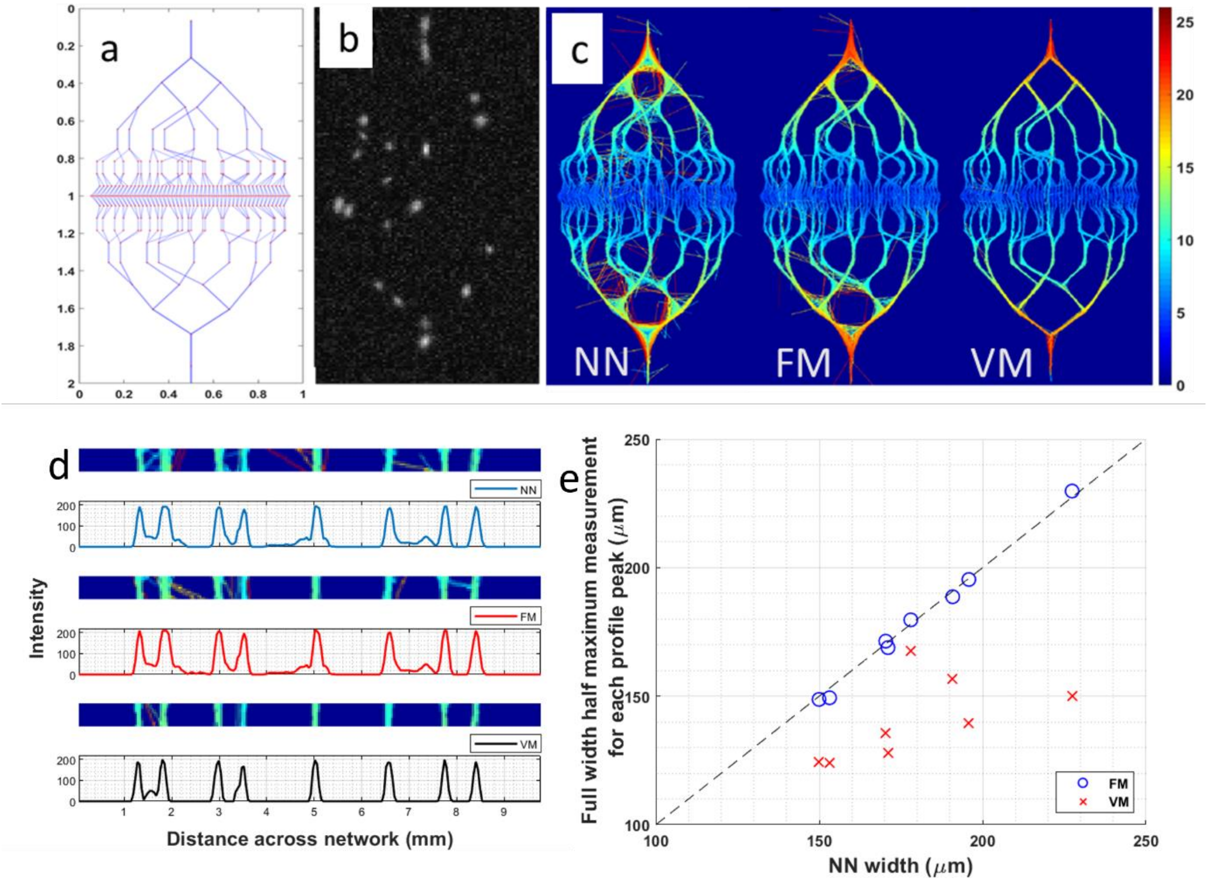

Fig. S2 (**a**) The simulated network structure, (**b**) an example individual frame of the synthetic contrast enhanced ultrasound video data (**c**) The microbubble speed maps calculated from the synthetic data set using nearest neighbour (NN), forward model (FM) and vascular model (VM) versions of the tracking algorithm. (**d**) Average intensity profile through the selected region in part **(c**) for each processing method. Each vessel is better resolved in the VM version compared to the NN and FM versions. (**e**) scatter plot comparing average full width half maximum (FWHM) for each peak in part (**d**). FM (circles) and VM (x) methods are compared to NN (x-axis). The FWHM calculated by NN and FM models are very similar (mean NN 179.6± 25 µm mean FM 179.0 ± 26 µm) (*p*=.5), following y=x, while the VM consistently model produces narrower vessels (mean 140.7± 16 µm) (*p*=.001).

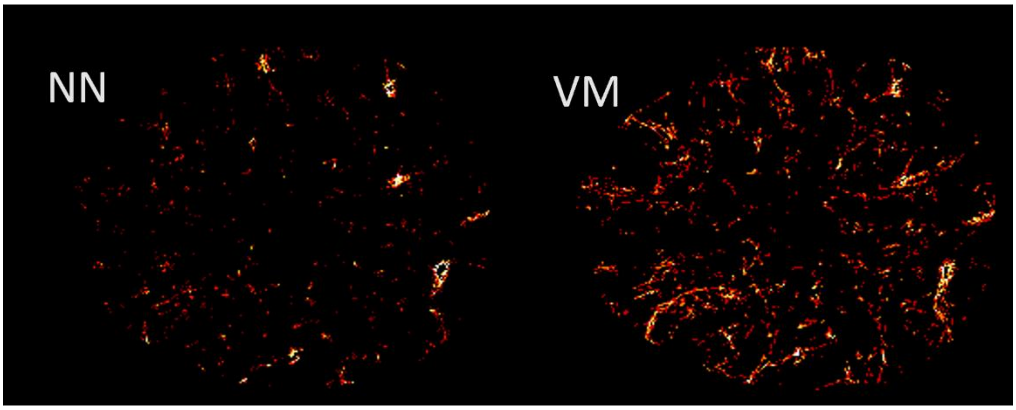

Fig. S3. Displaying 25-90% of the peak intensity value from the track number maps of the central Corpus Luteum (CL) region (see Figure 2) for Nearest Neighbour (NN) and Vascular model (VM) methods. The central CL is known to comprise very small vessels. The VM version of the code creates more links within this region and shows vessels which are likely to be between 200 and 50µm in diameter. The NN version produces few tracks and covers an area of 34 mm^2. The area covered inside the CL with VM is 82.2 mm^2, showing an increase of 137%.

Table S1 The specifications of the computational synthetic Vascular Network (detailed Figure S2 panel a). A tube-diameter range wider than 1.5 mm is provided which corresponds to tubes with realistic blood speeds ranging between 3.8 and 23.87 mm/sec.

Network vessel diameter (mm)	Average Tube length (mm)	Velocity [ground truth] (mm/sec)
0.5	0.5	3.8
0.63	0.6	6.6
0.794	0.7	8.1
1	1.5	11.5
1.26	1.7	14.8
1.587	2.2	18.7
2	2.6	23.87

Table S2 MRI and Ultrasound specifications[12] Abbreviations: AT = acquisition time, mpMRI = multiparametric magnetic resonance imaging, TR = Repetition Time, TE = Echo Time, FOV = Field of View. *(Invivo, Gainesville, FL, USA)

**MRI**			**Ultrasound**	
Scanner	INGENIA® MR System (Philips Medical Systems, Best, The Netherlands	MAGNETOM® Avanto (Siemens Healthcare, Erlangen, Germany	Scanner	Philips IU22 US scanner (Philips Healthcare, Bothell, USA)
Field strength and coils	3.0 Tesla<br>Pelvic phased array	1.5 Tesla<br>Pelvic phased array	Transducer<br>Prostate<br><br>Pre-clinical	 C10-3V endocavity<br><br>L9-3 Linear Array (both Philips Healthcare, Bothell, USA)
T2-Weighted:<br>• Sagittal<br>○ AT<br>○ Slice thickness (mm)<br>○ TR/TE (msec)<br>○ FOV (mm)<br>○ Pixelsize<br><br>• Transversal<br>○ AT<br>○ Slice thickness (mm)<br>○ TR/TE (msec)<br>○ FOV (mm)<br>○ Pixelsize<br><br>• Coronal<br>○ AT<br>○ Slice thickness (mm)<br>○ TR/TE (msec)<br>○ FOV (mm)<br>○ Pixelsize<br><br>DW-MRI:<br>○ Planes<br>○ AT<br>○ Slice thickness<br>○ TR/TE (msec)<br>○ FOV (mm)<br>○ Pixelsize<br>○ B-values	 00:05:09<br>3<br>4170/80<br>180<br>0.6 x 0.7<br><br>00:05:13<br>3<br>5801/100<br>200<br>0.5 x 0.6<br><br>00:03:39<br>3<br>3909/80<br>200<br>0.6 x 0.72<br><br>Transversal<br>00:06:26<br>3<br>3576/89<br>180<br>2.5 x 2.5<br>B50, B100, B1000, B1500<br>ADC = B50, 100 and 1000<br>Qualitative,	 00:05:22<br>4<br>4000/100<br>200<br>0.5 x 0.5<br><br>00:05:45<br>4<br>3500/100<br>200<br>0.5 x 0.5<br><br>00:04:18<br>4<br>3250/100<br>200<br>0.5 x 0.5<br><br>Transversal<br>00:03:29<br>5<br>4600/87<br>260<br>1.5 x 1.5<br>B50, B800<br>ADC	Patient<br>• Preparation<br>• Positioning<br><br>Contrast agent:<br>• Type<br>• Dosage<br>• Injections<br>• Record time<br><br>Pre-Clinical<br>• Type<br>• Dosage<br>• Data recording	IV-cannula<br>Lateral decubitus position<br><br>SonoVue® (Bracco, Geneva, Switzerland)<br>Bolus of 2.4mL SonoVue® with 5mL Saline flush<br>4 times: base, mid-base, mid-apex and apex<br>120 seconds from start bolus injection (primary study)<br>180 seconds continuing after the end of previous injection (Current study)<br><br>SonoVue® (Bracco, Geneva, Switzerland)<br><br>Bolus of 2.4mL SonoVue® with 5mL Saline flush<br><br>Video saved for duration of bolus injection, wash out frames (past peak intensity) used for processing.

○ Imaging sets<br><br>○ Quantitative analysis<br><br>DCE-MRI:<br>○ Planes<br>○ AT<br>○ Slice thickness (mm)<br>○ TR/TE (msec)<br>○ FOV (mm)<br>○ Pixelsize<br>○ Contrast agent (ml/sec)<br>Post-processing model	quantitative in unclear cases<br><br>Transversal<br>00:02:29<br>2<br><br>4.2/1.95<br><br>244<br>1.97 x 2.65<br>Gadovist (1.0 mmol/ml, 0.1 ml/kg) 2ml/sec<br>Tofts model using Dynacad*	Qualitative, quantitative in unclear cases<br><br>Transversal<br>00:02:00<br>4<br><br>50/3.9<br><br>320<br>2.2 x 1.6<br>Gadovist (1.0 mmol/ml, 0.1 ml/kg) 2ml/sec<br>Tofts model using Dynacad	US scanner settings:<br>Clinical<br>• Image<br>•<br>• Power-modulation<br>• Mechanical index<br>• Frame rate<br>• Focus<br>• Gain	Dual-image display: CEUS image and B-mode fundamental image<br><br>3.5 MHz<br><br>0.06<br><br>9-11 Hz<br><br>Positioned deep to achieve a uniform acoustic field of the prostate<br><br>Slightly above noise floor: 68-71%

Table S3 Synthetic data. Outputs comparing the detections and links made by 3 different versions of the tracking algorithm: Nearest Neighbour (NN), Forward Model (FM), Vascular Model (VM).

Synthetic	NN	FM	VM
Total links	17955	16559	13696
True positive (tp) links (percentage)	14542 (81.0 %)	15612 (94.3 %)	13136 (95.9 %)
False positive (fp) links (percentage)	3413 (19.0 %)	947 (5.7 %)	560 (4.1 %)
False negative (fn) links	6327	5257	7733
Jaccard index (%)	59.9	71.6	61.3
Precision (positive predictive value) = tp/(tp+fp)	81%	94.3%	95.9%
Recall (sensitivity) = tp/(tp+fn)	70%	74.8%	63%
$F_\beta = (1+\beta^2)\frac{prec \cdot rec}{\beta^2 prec + rec}, \beta = 0.5$	78.5%	89.6%	86.8%
Total tracks (links per track)	1441 (12.5)	1383 (12)	1849 (7.4)
Unused detections (percentage)	348 (1.8 %)	1803 (9.1 %)	4200 (21.3 %)

Table S4 Comparison of detection and linking outputs from contrast enhanced ultrasound video data from the sheep corpus luteum (CL) for nearest neighbour (NN) version and the Vascular model (VM) version.

Sheep CL Parameters	NN	VM
Frames processed	372 [136-507]	372 [136-507]
Detected MBs	17296	17296
Average MBs per frame	46	46
No of tracks	1798	1907
No of links	11375	8172
Average links per track	6.3	4.3
Percentage of unused detections	23.8%	41.7%
Mean velocity (standard deviation) (mm/s)	2.5 (1.1)	3.2 (1.9)
Median (quartiles 25% and 75%) (mm/s)	3.0 (1.7, 4.5)	2.5 (1.7,3.3)

Table S5 Quantitative output from Nearest Neighbour (NN) and Vascular Model (VM) tracking models from participant 127 Mid-Apex (Fig.3 main document)

P127 Parameter	NN	VM
Number of frames processed	1008	1008
Detected MBs	300260	300260
Average MBs per frame	298	298
No of tracks	23986	31169
No of links	230300	189950
Average links per track	9.6	6.1
unused detections (% unused)	45971 (15.3)	79144 (26.4)
Mean of velocities in speed map (Standard deviation) (mm/s)	1.65 (0.9)	2.1 (1.4)
Median velocities (quartiles) (mm/s)	1.9 (1.2, 2.6)	1.8 (1.1, 2.9)

Table S6 Processing outputs for P127 vascular model (VM) and nearest neighbour (NN) processing for cancer and non-cancer regions (ROI 1 and 2 in Fig 3)

P127 ROIs Parameter	NN Cancer ROI 1	VM Cancer ROI 1	NN No cancer ROI 2	VM No Cancer ROI 2
Number of frames processed	1008	1008	1008	1008
Detected MBs	11672	11672	6916	6916
Average MBs per frame	12	12	7	7
No of tracks	1297	1358	538	770
No of links	7462	4774	5472	4778
Average links per track	5.6	3.5	10.2	6.2
unused detections (% unused)	2913 (25.0)	5540 (47.5)	906 (13.1)	1368 (19.8)
Mean of velocities in speed map (Standard deviation) (mm/s)	3.6 (1.1)	4.1 (2.0)	2.8 (1.3)	3.1 (1.9)
Median velocities (quartiles25% and 75%) (mm/s)	3.7 (2.9, 4.3)	3.8 (2.6, 5.3)	2.7 (1.8, 3.6)	2.6 (1.6, 4.0)

Table S7 Output for both nearest neighbour (NN) and vascular model (VM) tracking models for P134 (Fig 4 main document). Also included are the numerical outputs (VM) from smaller regions of interest for cancer and non-cancer regions of the mid base plane.

P134<br><br>Parameter	NN	VM	Cancer ROI (VM)	Non Cancer ROI (VM)
Number of frames processed	1340	1340	1340	1340
Detected MBs	514410	514410	31003	29767
Average MBs per frame	384	384	23	22
No of tracks	47677	56340	3178	3166
No of links	361900	280770	21353	19623
Average links per track	7.59	4.98	6.7	6.2
unused detections<br><br>(% unused)	104840<br><br>(20.4)	177300<br><br>(34.5)	6472<br><br>(20.9)	6978<br><br>(23.4)
Mean of velocities in speed map (Standard deviation) (mm/s)	2.45<br><br>(1.1)	2.79<br><br>(1.7)	2.2<br><br>(1.4)	2.4<br><br>(1.5)<br><br>$p<0.001$ comparing to cancer region
Median velocities (quartiles 25% and 75%) (mm/s)	2.42<br><br>(1.6, 3.2)	2.46<br><br>(1.5, 3.8)	1.8<br><br>(1.1, 2.8)	2.1<br><br>(1.3, 3.1)

Table S8 Full summary of all clinical prostate data processing using super resolution ultrasound imaging. Each dataset which has been processed is represented and those where unusual regions of low, high or fast flow were noted (x). Entries in **bold** are associated with a clinical diagnosis of Gleason 7 cancer by biopsy. The super resolution method appears to have good sensitivity in that areas of faster flow can be identified and these are consistently associated with regions of cancer, further work is needed to fully assess the specificity and identify regions of the prostate where faster flow is normal.

	Participant (Overall diagnosis)	Imaging plane	Clinical Diagnosis (Systematic and targeted biopsy)	MRI diagnosis (Magnet strength)	SRI nature of identified suspicious regions		
					Avascular (low flow density or no flow)	High velocity or high flow density	High blood volume
1	122<br>(No PCa)	Base	No PCa	PIRADS 2<br>Benign<br>No PCa<br>(1.5T)			
2		Mid-Base	No PCa			x	
3		Mid-Apex	No PCa			x	
4		Apex	No PCa				
5	123<br>(T1cNxMx)	Base	Gleason 6	PIRADS 2<br>Benign<br>(3T)		x	
6		Mid-Base	Gleason 6				
7		Mid-Apex	Gleason 6		x		
8		Apex	No PCa				
9	124<br>(No PCa)	Base	No PCa	PIRADS 2<br>Benign<br>(3T)			
10		Mid-Base	No PCa			x	x
11		Mid-Apex	No PCa				
12		Apex	No PCa				

13	125<br>(No PCa)	Base	No PCa	PIRADS 2<br>Nothing suspicious<br>(1.5T)			
14		Mid-Base	No PCa				
15		Mid-Apex	No PCa				
16		Apex	No PCa				
17	126<br>No available diagnostic data	Base	PIRADS 4<br>Suspicious MRI Apex - peripheral zone - right<br>but no biopsy undertaken to confirm				
18		Mid-Base				x	x
19		Mid-Apex			x	x	
20		Apex				x	x
**21**	**127**<br>**(T2cNxMx)**	**Base**	**Gleason 7**	**PIRADS 5**<br>**Suspicious in the left base - peripheral anterior zone**<br>**(1.5T)**	**x**		
**22**		**Mid-Base**	**Gleason 7**		**x**		
**23**		**Mid-Apex**	**Gleason 7**		**x**	**x**	**x**
24		Apex	No slice available				
25	128<br>**(T1cNxMx)**	Base	No PCa	**PIRADS 4 Mid prostate left posterior, lateral peripheral zone**<br>**(3T)**		x	
**26**		**Mid-Base**	**Gleason 7**			**x**	
**27**		**Mid-Apex**	**Gleason 7**		**x**	**x**	
28		Apex	No PCa		x		

29	129 (No PCa)	Base	No slice available	PIRADS 2 Benign (3T)			
30		Mid-Base	No PCa		x	x	
31		Mid-Apex	No PCa		x		
32		Apex	No PCa				
33	130 (No PCa)	Base	No PCa	PIRADS 2 Benign (3T)			
34		Mid-Base	No PCa				
35		Mid-Apex	No PCa				
36		Apex	No PCa				
37	131 (No PCa)	Base	No PCa	PIRADS 2 Benign (1.5T)			
38		Mid-Base	No PCa				
e		Mid-Apex	No PCa				
40		Apex	No PCa				
41	132 (No PCa)	Base	No PCa	PIRADS 2 Benign (3T)			
42		Mid-Base	No PCa				
43		Mid-Apex	No PCa		x		
44		Apex	No PCa			x	x
45	133 (T2cNxMx)	Base	Benign Gleason 6	PIRADS 2 Benign (3T)		x	x
**46**		**Mid-Base**	**Gleason 7**		**x**	**x**	
47		Mid-Apex	Gleason 6				
48		Apex	No PCa				
49	**134 (T2cNxMx)**	Base	Gleason 6	**PIRADS 4 Mid prostate**			
**50**		**Mid-Base**	**Gleason 7**		**x**	**x**	**x**
51		Mid-Apex	No PCa			x	

52		Apex	No PCa	**Left posterior medial**<br>**(3T)**		x	x
**53**	**135**<br>**(T3aNxMx)**	**Base**	**Gleason 7**	**PIRADS 5**<br>**Base right posterior, lateral,**<br>**Mid prostate posterior lateral**<br>**(1.5T)**	**x**		
**54**		**Mid-Base**	**Gleason 7**		**x**	**x**	**x**
**55**		**Mid-Apex**	**Gleason 7**		**x**	**x**	
56		Apex	No PCa				x

Mapping of prostate cancer microvascular patterns using super-resolution ultrasound imaging – supplemental text

Introduction

The following supplemental material covers the methods and results for the *in silico* work as well as further details on the processing of the ultrasound video data. Also included is further clinical data in the form of a summary of all processed prostate data along with further results related to the in silico, pre-clinical and clinical data processing presented in the main manuscript.

Methods

In silico methods

Initial development of the tracking model was undertaken using a computational in-silico vessel network with synthetic ultrasound image data, which enabled the testing of different versions of the algorithm in a quantitative manner.

Synthetic data generation

The fluid flow model is based on the Navier-Stokes equations, and this process requires significant computational power. Thus, it is simplified, assuming boundary conditions, to fluid flow in tubes. The simplified problem is described by the Hagen-Poiseuille equation:

$$\Delta P = 8\mu LQ/(\pi R^4) \qquad \text{(Eq. 1)}$$

where: ΔP is the pressure difference between the two ends, L is the length of pipe, μ is the dynamic viscosity, Q is the volumetric flow rate and R is the tube radius. Using the above, flow networks were constructed simulating blood flow in a vascular space. The network structure is displayed in Supplemental Fig. S2 and comprises different tubes that connect to one another with varying patterns following Murray's Law. Murray's Law for bifurcation determines the relation between the diameters of a parent (R_i) and child vessels ($R_{i,1}$, $R_{i,2}$) with the expression: $R^3_i = R^3_{i,1} + R^3_{i,2}$. The dynamic viscosity of the fluid was set equal to that of blood and realistic human blood velocities [1-3] were implemented.

Based on the above, the moving MBs within the vascular network are simulated. The position within the fluid is sampled, and the spatial coordinates of dimensionless particles recorded. These coordinates correspond to coordinates of particles moving with the same flow parameters. Each particle has a specific ID while the physical properties of the particles are not simulated. Finally, the particle coordinates are the MB locations at the US frames.

The synthetic US data are generated using an US image simulator that, instead of deploying a widely available software (e.g., Field II), is tailored to realistically reflect the MB echo image appearance and morphology and is extracted from real CEUS data. This way any near-field aberration, artefacts and the MB nonlinear response resulting in an increase in point spread function variability across the image, and cannot be simulated using Field II, need not to be considered as the MB images are retrieved from real data. The process from the selection of a real frame sequence to the generation of the synthetic vessel network with moving MBs is as follows.

A video loop from real CEUS data from the sheep ovary is chosen. This has high SNR and includes a high proportion of well separated single MB echoes. The MB detection and segmentation algorithm is applied to extract images of multiple MBs in every frame. MBs are then fitted to 3D Gaussian distributions which enables the extraction of a number of parameters including intensity, size in all coordinates, orientation, and goodness of fit in terms of the root-mean-squared-error (RMSE) for every fit. The synthetic ultrasound frames are assigned a number of randomly selected MBs which are available within the distribution.

Synthetic vascular network

The synthetic vascular network presented here simulates the motion of the MBs within vessels with diameters ranging from 0.5-2 mm, as depicted in Table S1. The construction of the computational network was based on the real geometry relationship between parent and child vessels (bifurcation or trifurcation) and the motion of MBs was based on fluid flow modelling [4, 5].

The position of the fluid was sampled, and the spatial coordinates of dimensionless particles were recorded according to the adopted fluid model. These coordinates correspond to particles moving with the same flow parameters in a single line through the centre of each vessel within the network. Each particle was assigned an 'ultrasound image' from the compiled synthetic ultrasound data. The coordinates of the particles within the network were saved and used as the ground truth location for the MBs.

Super Resolution Ultrasound Tracking methods

The tracking algorithm is based on detection, localization and tracking of echoes from MBs. Current limited research in this area employs a Markov Chain Monte Carlo (probabilistic) method [6], or a Kalman filter-based approach [7] to account for direction and acceleration constraints. There is little evidence that the adopted methods perform well on real world data and the underlying knowledge of vascular dynamics is not deployed. The tracking algorithms considered were described in the main manuscript. For the vascular model (VM) Criteria for rejecting links include assessment of the average MB path density. Links which have significantly different MB densities at the start and end points of the track, or links whose MB path density has a large deviation compared to its average value. Example of such links are shown in Supplemental Fig S1 where a true link is circled black with consistent MB density and rejected links circled white where the link is made but the MB density has high variability along the path. These additional rejection criteria ensure that tracked MBs stay within the vascular space and do not jump across different vessels.

Pre-clinical methods

The animal work was conducted under home office licence Duncan PPL 60/4401. Contrast enhanced ultrasound videos of ovine ovaries were saved following published protocols [8]. The ovaries were exposed via laparotomy, and one at a time brought to the surface and positioned in order to retain full blood flow, a layer of ultrasound gel provided protection for the ovary as well as good contact for the ultrasound. A Philips iU22 ultrasound scanner with linear array probe L9-3 was secured over the ovary, imaging through the maximum diameter cross-section of the CL. SonoVue (Bracco), an ultrasound contrast agent, was administered by 2.4 mL bolus injections. Various imaging parameters (such as image depth and image

zoom) were assessed in order to optimise the real-life ultrasound imaging requirements for optimal video data. Both ovaries in 4 different sheep were assessed. The videos of the contrast intensity wash-in and wash-out were saved and processed offline.
After ultrasound imaging the ovary vessel cell walls were stained with 70-μL rhodamine labelled Griffonia (Banderiaea) simplicifolia lectin 1 (GSL 1 lectin; Vector Labs, United Kingdom). The ovary was removed, stored in 4% paraformaldehyde and processed for imaging with Optical Projection Tomography (OPT). Images were acquired using a Bioptonics 3001 OPT scanner (Bioptonics, Edinburgh, United Kingdom) the ovary tissue was auto-fluorescent in the green channel and GSL 1 labelled vessels were imaged in the red channel. The OPT outputs were saved as images and used to create a 3D reconstruction of the ovary. A slice through the OPT data set in a location as close as possible to the ovary was identified for comparison with the ultrasound data. Features and vessels seen in the super resolution ultrasound imaging maps were matched with those seen in the OPT image and large vessels seen in the ultrasound imaging maps were identified on the OPT image.

Clinical methods

In addition to the details in the main manuscript, details of the MRI and Ultrasound specifications are given in supplemental Table S2.

Pre-processing of contrast enhanced ultrasound data

For both pre-clinical and clinical data, the original ultrasound video was imported into ImageJ software, cropped around the region of interest (ROI), and saved as an image sequence. For pre-clinical data the ROI of the displayed data was the boundary of the CL within the ovary and for the clinical data the ROI was the boundary of the whole prostate as guided by B-mode ultrasound. The pre-processing of the cropped images included registration (sheep data [9]), and removing pixels with low intensity display values has been previously described [10, 11].

Results

Synthetic data

Supplemental Fig. 2 (panel a) depicts the design of the in silico vessel network and Fig. S2 (panel b) an example image frame from the synthetic ultrasound data applied to the same network. The localization accuracy of the detection method applied to the synthetic ultrasound data was assessed by comparing the known ground truth centre with the localised centre of the detection and was found to be 25.8 μm (λ/20). When detections were located close together and eventually overlapping the options were to try to separate the

detections or treat it as a single large detection. When overlapping MBs were treated as multiple separate detections and assessed in terms of distance from the centre of the vessel, a deviation of 159 ± 103 µm was found compared to 20.5 ± 11.6 µm to when a single (large) detection was assumed thus showing that the single detection approach best represents the vascular structure. Speed maps produced by each version of the algorithm tested (NN, FM, VM) are shown in Fig. S2 (panel c). The NN-based linking shows multiple incorrect links between unconnected vessels (e.g, yellow arrow); FM shows improvement with some of the extra tracks being filtered out, but incorrect links are still observed. Linking and velocity estimation is optimised in the VM model. Fig. S2 (panel d) represents the mean intensity profile across a section of vessels in Fig. S2 (panel c) (profile region marked by rectangle) and represents the resolution across a vessel. The full width half maximum (FWHM) values for each peak in the profiles in Fig. S2 (panel d) were calculated and are displayed in the scatter plots in Fig. S2 (panel e) where both the FM and VM resolution is compared to the NN resolution. The scatter plot clearly shows that the FM and NN produced similar values for the FWHM (circles) while the VM model produced narrower peaks and therefore better resolution. The Shapiro-Wilks test statistic (W) was applied to the calculated FWHMs(n=8) with outputs for NN (W 0.938 p=.590), FM (W 0.932 p=.529) and VM (W 0.915 p=0.395). For each algorithm p>.05 therefore normality is assumed and t-tests were applied to determine significance. The FWHM calculated for NN and FM models are very similar (Fig. S2 (panel e)) (mean NN 179.6.7± 25 µm, mean FM 179.0 ± 26 µm) (p=.5), while the VM model produces consistently smaller values (mean 140.7.4 ± 16 µm) (p=.001 comparing VM with FM and NN). The profiles in Fig S2 (panel) also show that while most of the peaks are well defined in the VM model some low intensity false peaks remain. In addition, there is distortion in the reconstruction of the bifurcations in NN and FM while well-defined bifurcations are seen with VM (Fig. S2 panel c). Numerical outputs of number of tracks and links for each model are provided in the supplemental material (Supplemental Table S3). Supplemental Table S3 shows the associated numbers of tracks and detections made using each version of the linking model applied to the synthetic dataset. Important observations for the VM model include fewer links per track with a higher total number of tracks and low number of incorrect links (4.1%) compared to the FM (5.7%) and the NN (19%). As a consequence the reconstructed vessels maps are narrowest for the VM, which also results in improved velocity estimation, even at the bifurcations. Another consequence of the VM model is the reduction in false links, leading to an increase in the number of unused MB detections (21.3% compared to 9.1% in the FM model), which also results in a reduction of Jaccard index from 71.6% for the FM to 61.3% in the VM model. For all versions the minimum number of links per track is 3.

Pre-clinical

The application of the NN and VM algorithms to pre-clinical sheep data shows similar trends to the synthetic data. Supplemental Table S4 displays the detection and linking outputs for an example data set. Again, there are fewer links but more tracks for the VM version compared to the NN version along with shorter tracks and more unused detections in the VM version. The VM model provides more consistent tracks, with higher average velocity. This is a consequence of the higher percentage of unused detections at 42% for the VM compared to 24% for the NN model and fewer links per tracks with VM. These results along with the qualitative assessment verifies that better resolved vessels with more vascular structure are formed with the VM model, while using less data than the NN model. Along with the improvement of resolution the overall consistency with the synthetic data strongly suggests that the erroneous vessel creation is insignificant for the VM model while the NN model fails to show well defined vessels. Particularly, Fig S3 shows smaller vessels inside the CL are better captured with the VM, occupying an area of 82.2 mm^2 in contrast to NN, which occupies and area of 34 mm^2. Therefore, the VM achieves an increase of 137% in the area covered inside the CL which corresponds to the smaller vessels. An example of super-resolution maps of the CL created using NN and VM as well as the associated OPT image confirming the location of the larger vessels surrounding and feeding the CL are presented in the main manuscript.

Clinical

Super resolution maps created from processing clinical ultrasound video data of prostate cancer are given in the main document. Numerical assessment of the outputs from processing is provided here. Outputs from processing of the mid-apex slice of participant 127 using both NN and VM methods are presented in Supplemental Table S5 showing a lower average number of links per track and a larger number of unused detections for the VM model compared to NN due to NN creating more false links as shown with the synthetic data. Supplemental Table S6 compares the outputs for the 2 smaller regions within the same prostate slice. Region 1 is the known cancer region and region 2 is a region on the same side of the prostate which is not thought to contain any cancer regions (from biopsy). Both regions use the same size ROI and the locations are identified in the manuscript Fig 3. For both VM and NN there were more detections and tracks created in the cancer region compared to the non-cancer region. More unused detections were seen in the cancer region, perhaps due to the increased number of detections and density of the contrast. For both ROIs, comparing NN and VM processing gives more tracks from the VM processing but containing fewer links. Both processing methods gave faster velocities in the cancer region compared to the non-cancer region. It should be noted that the cancer region on the left

side, found by biopsy, was not assessed in the same way due to uncertainty in the prostate boundary and cancer location. T-tests were undertaken to compare the outputs from the different regions and are provided in the main document.

Numerical outputs from processing of the mid-base slice for participant 134 using VM and NN are presented in Supplemental Table S7. Similar trends are seen in both VM and NN versions of the processing, however the VM continues to produce more tracks with fewer links compared to the NN model. Unlike P127, for P134 the cancer region contained tracks of slightly slower velocity compared to the non-cancer region ($p<.001$).

Supplemental Table S8 provides the list of all prostate data with their clinical diagnostic information along with the vascular features noted in the SRI processed maps. 15 prostate imaging planes were found to have avascular regions with many of these located beside a region of high or fast flow. 20 slices contained regions of high velocity (compared to the rest of the same prostate slice) or high-density flow while 10 slices had regions of high blood volume. Of the 10 imaging planes (from 5 participant cases) with confirmed Gleason 7 cancer 9 contained avascular regions, 7 had regions of fast and dense flow while 6 had both avascular and high flow regions.

Some regions of high or fast flow were noticed in participants that appear not to have significant cancer, however in general participants with no known cancer have low flow velocities in sparse distributions of vessels and may potentially be well differentiated from participants with Gleason 7 cancer. These observations may improve diagnostic sensitivity and negative predictive value in detecting significant cancer. However, a future study design may include improved location identification in ground truth data, such as fusion or post-op histology, in order to validate all features associated with different prostate cancer presentations. , in short of the 10 imaging planes with confirmed Gleason 7 cancer, 9 contained avascular regions, 7 had regions of fast and dense flow while 6 had both avascular and high flow regions at the assumed cancer region from the clinical data.

Discussion/summary

The supplemental material gives details on the development of SRUI tracking algorithms which have been assessed and eventually applied to clinical prostate cancer data. Initial algorithm development was undertaken on synthetic data in and in silico vessel network in order to develop a reliable and well-suited model. The current best model is described as the VM model where many detected contrast echoes are not utilized in the MB pairing but provides outputs in which there is high confidence. The final summary of the clinical data shows that the method is sensitive in that many image planes were found to have regions of faster blood flow or regions with low density flow leading to the conclusion that SRUI

processing in prostate cancer is highly sensitive, but more clinical data is needed to inform on the specific features associated with prostate cancer and thus increase specificity.

Supplemental References

1. K.P. Ivanov, M.K. Kalinina, Y.I. Levkovich (1981) Blood flow velocity in capillaries of brain and muscles and its physiological significance, Microvascular Research 22:143−155. https://doi.org/10.1016/0026-2862(81)90084-4

2. M. Kobari, F. Gotoh, Y. Fukuuchi et al (1984) Blood flow velocity in the pial arteries of cats, with particular reference to the vessel diameter, Journal of Cerebral Blood Flow & Metabolism 4:110−114. https://doi.org/10.1038/jcbfm.1984.15

3.F. Martini, J.L. Nath, E.F. Bartholomew (2008) Fundamentals of Anatomy & Physiology, Pearson/Benjamin Cummings.

4. A. Boujelben, M. Watson, S. McDougall et al (2016) Multimodality imaging and mathematical modelling of drug delivery to glioblastomas, Interface Focus 6:20160039. https://doi.org/10.1098/rsfs.2016.0039

5 T.F. Sherman (1981) On connecting large vessels to small. The meaning of Murray's law, J Gen Physiol 78:431−453. https://doi.org/10.1085/jgp.78.4.431

6. D. Ackermann, G. Schmitz (2016) Detection and tracking of multiple microbubbles in ultrasound B-mode images, IEEE Transactions on Ultrasonics, Ferroelectrics, and Frequency Control 63:72−82. https://doi.org/10.1109/tuffc.2015.2500266

7. S. Tang, P. Song, J.D. Trzasko et al (2020) Kalman ilter-based microbubble tracking for robust super-resolution ultrasound microvessel imaging, IEEE Transactions on Ultrasonics, Ferroelectrics, and Frequency Control 67:1738−1751. https://doi.org/10.1109/tuffc.2020.2984384

8. V. Sboros, M. Averkiou, M. Lampaskis, et al (2011 Imaging of the ovine orpus luteum microcirculation with contrast ultrasound, Ultrasound in Medicine & Biology 37:59−68. https://doi.org/10.1016/j.ultrasmedbio.2010.10.013

9. A. Perperidis, D. Thomas, M. Averkiou, et al (2014) Automatic dissociation between microvasculature and larger vessels for ultrasound contrast imaging, 36th Annual International Conference of the IEEE Engineering in Medicine and Biology Society, 5076−5079. https://doi.org/10.1109/embc.2014.6944766

10 E. Kanoulas, M. Butler, C. Rowley et al (2019) Super-resolution contrast-enhanced ultrasound ethodology for the Ideintification of in vivo vascular dynamics in 2D. Invest Radiol. 54:500−516. https://doi.org/10.1097/rli.0000000000000565

11. M. Butler, A. Perperidis, J.-L.M. Zahra, et al (2019) Differentiation of vascular characteristics using contrast-enhanced ultrasound imaging, Ultrasound in Medicine and Biology 45:2444−2455. https://doi.org/10.1016/j.ultrasmedbio.2019.05.015

12. C.K. Mannaerts, M.R.W. Engelbrecht, A.W. Postema, et al (2020) Detection of clinically significant prostate cancer in biopsy-naïve men: direct comparison of systematic biopsy, multiparametric MRI- and contrast-ultrasound-dispersion imaging-targeted biopsy, BJU International 126:481−493. https://doi.org/10.1111/bju.15093
